# A new methodology for evaluating the neighbor discovery time in schedule-based asynchronous duty-cycling wireless sensor networks

**DOI:** 10.1016/j.mex.2024.102967

**Published:** 2024-10-10

**Authors:** Diego Passos, Beatriz Trabbold, Ricardo C. Carrano, Cledson de Sousa

**Affiliations:** a Departamento de Engenharia Eletrónica e Telecomunicações e de Computadores, Instituto Superior de Engenharia de Lisboa (ISEL), Lisbon, Portugal; b Departamento de Engenharia de Telecomunicações, Escola de Engenharia, Universidade Federal Fluminense, Niterói RJ, Brazil

**Keywords:** Wireless sensor networks, Duty cycling, Neighbor discovery time, Subslot-based Neighbor Discovery Time

## Abstract

Duty cycling is a fundamental mechanism for battery-operated wireless networks, such as wireless sensor networks. Due to its importance, it is an integral part of several Medium Access Protocols and related wireless technologies. In Schedule-based Asynchronous Duty Cycle, nodes activate and deactivate their radio interfaces according to a pre-designed schedule of slots, which guarantees overlapping uptime between two neighbors, independent of the offset between their internal clocks, making communication between them possible. This paper presents a new methodology for evaluating the Neighbor Discovery Time (NDT) of Schedule-based Asynchronous Duty Cycle. Differently from previous methodologies, it accounts for the possibility of the slots in the schedules of the two neighbors not being perfectly border-aligned — an unrealistic assumption in practice. By means of simulation, we show that not taking this under consideration can lead to an overestimate of the NDT by a factor of 2 depending on the particular scenario, thus justifying the importance of our work.

• We propose a new subslot-based methodology for computing the NDT of a wakeup schedule used for asynchronous duty cycling.
• It replaces the traditional slot-based methodology, by dividing slots into subslots, allowing for the analysis of non-integer clock offsets between nodes, and further allowing mathematical models to consider the more realistic continuous-time case.
• Our validation data shows that the slot-based methodology may overestimate NDT by a factor of up to 2, making the proposed subslot-based methodology much more precise.

## Graphical abstract

**Figure:**
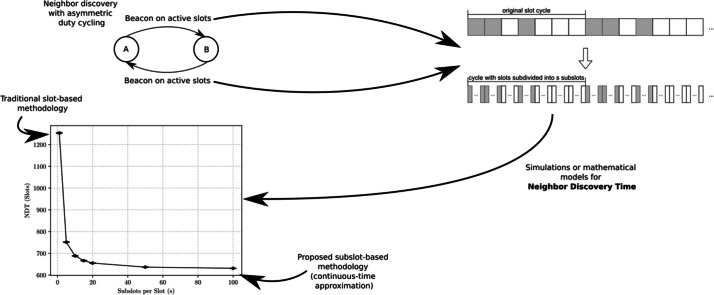

Specifications table

**Table:** 

Subject area:	Computer Science
More specific subject area:	Computer Networks
Name of your method:	Subslot-based Neighbor Discovery Time
Name and reference of original method:	Carrano, R. C., Passos, D., Magalhaes, L. C., & Albuquerque, C. V. (2014). A comprehensive analysis on the use of schedule-based asynchronous duty cycling in wireless sensor networks. Ad Hoc Networks, 16, 142–164.
Resource availability:	https://github.com/diegogpassos/subslotNDT

## Background

In Wireless Sensor Networks (WSNs), nodes often operate under strict energy constraints and lifetime is limited by the autonomy of batteries [8]. Since the radio is one of the most power-hungry components of a mote, duty cycling the network interface becomes a necessity [1]. Duty cycling consists of turning the radio off whenever it is not needed. This is done by introducing some kind of coordination between nodes so that neighbors are still guaranteed to have *encounter opportunities*.

One approach is the so-called *schedule-based asynchronous duty cycling*, which uses specially crafted wakeup schedules defined as a cyclically pattern of discrete slots, which can be either active (radio on) or inactive (radio off). If two unsynchronized nodes follow the same schedule, that is equivalent to one of those nodes following a rotation of original schedule – the number of slots by which the schedule is rotated corresponds to the offset between the nodes’ internal clocks. Thus, schedules must guarantee at least one common active slot between itself and any of its rotations, which is known in the literature as the *rotation closure property* (RCP).

Several algorithms can yield schedules with RCP and arbitrarily low duty cycles – the fraction of active slots. Among the classical strategies, one can cite Grid, Torus, Disco, Block Designs [3], Uconnect [5], and Searchlight [2]. For instance, Grid creates schedules with *n*^2^ slots, of which 2n−1 are active. By increasing n, the duty cycle – $\frac{2n-1}{n^{2}}$ – decreases monotonically.

There is a tradeoff between the duty cycle of a schedule and its Neighbor Discovery Time (NDT) [4], *i.e.*, the expected number of slots from the moment a node decides to communicate with a certain neighbor until the corresponding packet is successfully received, considering all possible offsets and starting slots. For a given method, schedules with a lower duty cycle result in higher NDT, but certain methods are known to generate more efficient schedules – *i.e.*, with a better tradeoff between NDT and duty cycle [4]. Therefore, accurately measuring the NDT of a schedule is fundamental for evaluating schedule-based duty cycling methods.

Currently, NDT is computed by considering only integer offsets, allowing both simulations and mathematical models to only average a limited number of cases, a methodology proposed in [4] and hereinafter called the *slot-based methodology*. However, an integer offset implies any two neighbors have their slots border-aligned, which is highly unlikely.

In this paper, we present a *subslot-based methodology* for evaluating the NDT considering non-integer offsets. We present a case study of applying our methodology to Block Designs and show how it can be used to derive models considering the offset as a continuous random variable, instead of a discrete one – a common simplification found on the literature. We validate our methodology by means simulations and show that the slot-based methodology can overestimate NDT by a factor up to 2.

## Method details

Several works in the literature evaluate the NDT of asynchronous duty-cycle schedules by means of either numerical simulators or mathematical models [6,7]. Perhaps the seminal work to explore these approaches was [4]. In essence, both approaches compute an average considering all possible combinations of rotations between the schedule and itself, as well as the starting slot for the transmitting node – *i.e.*, the slot in which the transmitting node decides to communicate with its neighbor. For a given combination of rotation and starting slot, the model or simulator counts the number of slots until the first encounter opportunity. Given a schedule with a length of n slots, there are $n^{2}$ such combinations.

Some models and simulators are simplified and instead consider only a subset of those combinations, thus yielding an approximation for the NDT. Among those, *probabilistic simulators* perform a certain number of repetitions, which consist of randomly choosing a rotation and starting slot, and obtaining a sample for the NDT. Later, those samples are averaged to obtain an approximation for the NDT. Those probabilistic simulators are particularly useful in cases where one wishes to also take into account a non-zero frame loss probability – *i.e.*, when multiple encounter opportunities may be needed because the transmitted frames can be lost.

### Dividing the schedule into subslots

In the subslot-based methodology we present in this paper, we consider an extension of these original approaches. The basic idea is to consider that the rotations between two nodes’ schedules can be by a non-integer number of slots. Indeed, that is the most likely case in practice, as the phenomena that causes nodes to be unsynchronized – *e.g.*, clock drift, different power-on times – can cause any time offset between their clocks, and not only integer multiples of the slots length.

The challenge, however, is that there are infinite many possible non-integer offset values. That makes the usual approach of simulators and mathematical models that exhaustively consider all possible combinations of offsets and starting slots unfeasible. Furthermore, even the probabilistic simulators, that only consider a sample of the possible combinations, would require much more repetitions for providing statistically significant results.

**Fig. 1:**
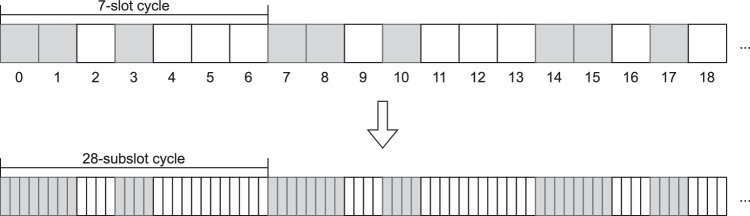
Mapping from an original {7,3,1} block-design schedule to the augmented schedule with slots subdivided into slots. For this example, we used *s* = 4.

Instead, we propose a middle ground between those two approaches: for computing the NDT, we divide each slot into s subslots, as illustrated in Fig. 1. The original schedule shown in the figure corresponds to a few repetitions of a {7,3,1} Block Design – each cycle is composed of 7 slots, 3 of which are active, and it guarantees at least 1 common active slot with any rotation of itself. In the bottom of the figure, we show the same schedule but now with each original slot subdivided into s=4 subslots.

The resultant augmented schedule can be analyzed as any other schedule and, in particular, its NDT can be computed by any of the traditional approaches. By remembering that the resultant NDT is computed in subslots and performing the appropriate conversions – in this example, by dividing the result by s=4 –, one can arrive at the NDT for the original schedule, but now accounting for any offset that is a multiple of $\frac{1}{4}=0.25$ slots. By increasing the number of subslots, we can increase the resolution of the NDT computation at the expense of having to cope with larger schedules.

### Subslots and beacon transmissions

The main application for schedule-based asynchronous duty cycle methods is low-power neighbor discovery. Under this application, a sensor sends beacon frames in every active slot. Depending on the particular discovery protocol, it can also involve the exchange of other frames, such as an ack. The traditional slot-based model simply assumes that both the transmission of the beacon and its respective reception by the neighbor node must occur over the same active slot and, therefore, an encounter opportunity happens when the two neighboring sensors have a common active slot. Under this hypothesis, details of the protocol used during an active slot do not matter.

However, those details do matter when we consider the subslot division of the subslot-based methodology, because now each slot is replaced with s subslots. If we simply treat each subslot exactly as we would treat an original slot, we would be implicitly assuming many more beacon transmissions and, therefore, we would arrive at a unfeasibly low value for the NDT.

Because of that, in the proposed subslot-based methodology we assume that a sensor only transmits a single beacon at the beginning of each slot and spends the rest of the slot listening for incoming frames. Notice that this assumption can be easily adapted to different implementations that eventually transmit more beacons per slot or that transmit the beacons on other parts of the slot (*e.g.*, at the end).

Given the assumption that beacons are only transmitted at the beginning of a slot, the definition of an encounter opportunity must be altered. More specifically, an encounter opportunity happens when the following two conditions are met simultaneously:

• the transmitting node is in the first subslot of an active slot; and
• the neighbor node is in any subslot of an active slot.

**Fig. 2:**
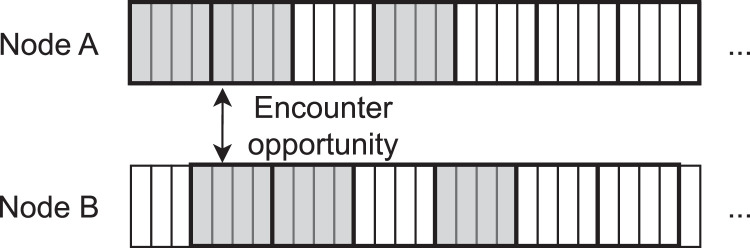
Example of the definition of an encounter opportunity considering the proposed subslot-based methodology.

Fig. 2 illustrates this alternative definition of encounter opportunity. In the example, two nodes A and B follow a {7,3,1} Block Design but with an offset of 0.75 slots – *i.e.*, 3 subslots using s=4. Despite both A and B being active simultaneously in several subslots, only on the 5th subslot shown in the figure they are active at the same time and A is in the first subslot of a slot (*i.e.*, transmitting a beacon).

## Unidirectional *vs.* bidirectional discovery

Another factor that is not directly relevant to traditional slot-based methodology but affects the output of the subslot-based methodology is whether the discovery scenario is unidirectional or bidirectional. In this context, unidirectional and bidirectional refer to whether only one of the nodes transmits beacons or if they both do.

Consider, for example, a setup phase of a WSN in which all sensors are deployed approximately at the same time and spend the first minutes of operation trying to discover their neighbors. Because all nodes are simultaneously in this discovery mode, they will all transmit beacons at the beginning of their respective slots. If we focus on two nodes, A and B, as illustrated on Fig. 2, this means that encounter opportunities will happen if one of the following conditions is met:

• A is in the first subslot of an active slot and B is in an active subslot; or
• B is in the first subslot of an active slot and A is in an active subslot.

On the example of Fig. 2, that means that the 4th subslot would also be an encounter opportunity, as B is in the first subslot of its active slot and A is an active subslot as well. In total, 4 encounter opportunities are shown in Fig. 2 under this criterion – 3 of which happen due to beacons transmitted by B and 1 for a beacon transmitted by A. We refer to this as the *bidirectional discovery case*.

Now, consider a different scenario in which the network has been operating for some time and, therefore, is not in the setup phase. Assume that a new sensor A is then deployed. Because A just entered the network, it needs to discover its neighbors and, therefore, it operates during the first minutes in the discovery mode, transmitting beacons at the beginning of each of its active slots. However, the remaining nodes do not transmit beacons anymore.

Under those assumptions, there will be a smaller number of encounter opportunities, because those will require A to be in the first subslot of its active slots. That is illustrated in Fig. 2 which shows a single such opportunity. We refer to this as the *unidirectional discovery case*.

Because the unidirectional discovery case yields less encounter opportunities, the resultant NDT is lower under the bidirectional case. Notice that this differentiation between the unidirectional and bidirectional cases does not directly impact the slot-based methodology, as there is no differentiation of the active slots. However, for the proper application of proposed subslot-based methodology, it is important to correctly identify which discovery case is used.

### Applying the proposed methodology: simulations

The simplest application of the subslot-based methodology is in the form of a numerical simulator. The simulator receives as inputs the schedule followed by the two simulated nodes, A and B, the number of repetitions to perform, the probability p of a successful discovery when a node transmits a beacon and the receiver is active, the number s of subslots to divide each slot into, and the discovery mode (unidirectional or bidirectional). It then performs the following steps:

1. Generate the augmented schedule by subdividing each slot into s subslots.
2. Execute a repetition of the simulation:
  a. Randomly select an initial slot for node A.
  b. Randomly select an offset between the internal clocks of A and B.
  c. If the discovery mode is bidirectional:
    i. Find the next subslot for which both A and B are active and A is in the first subslot of a slot.
  d. Else:
    i. Find the next subslot for which both A and B are active and either A or B is in the first subslot of a slot.
  e. Determine if the discovery was successful by drawing from a random variable X uniformly distributed in the interval [0,1] and checking if X≤p.
  f. If the discovery was not successful, return to step 2c.
  g. Store the number of subslots elapsed since the beginning of this repetition in an array of samples.
  h. If the requested number of repetitions was reached, go to step 3.
  i. Else, go back to step 2.
3. Output the estimated NDT by averaging the samples stored on the array and dividing the result by s.

For validating the subslot-based methodology and understanding how its estimated NDT values differ from those obtained with the traditional slot-based methodology, we implemented a simulator following those steps, which is available along with the resources that complement this paper.

### Applying the proposed methodology: mathematical models

Aside from being used in numerical simulators, the proposed subslot-based methodology can also be used to derive mathematical models for predicting the NDT for certain schedules or for schedules generated by a certain algorithms. That is done, for example, in [4], but considering the traditional slot-based methodology. Instead, we can obtain more accurate mathematical models by considering the subslot-based methodology.

We illustrate that by considering Block Designs as a case study. In the context of asynchronous duty cycling schedules, a {v,k,λ} Block Design is a specially crafted wakeup schedule that has a cycle length of v slots, k of which are active, while guaranteeing exactly λ common active slots per cycle for any (integer) offset different from 0. When λ=1, Block Designs are known as Projective Planes and correspond to the shortest possible schedules that guarantee the RCP for a given duty cycle value.

**Fig. 3:**
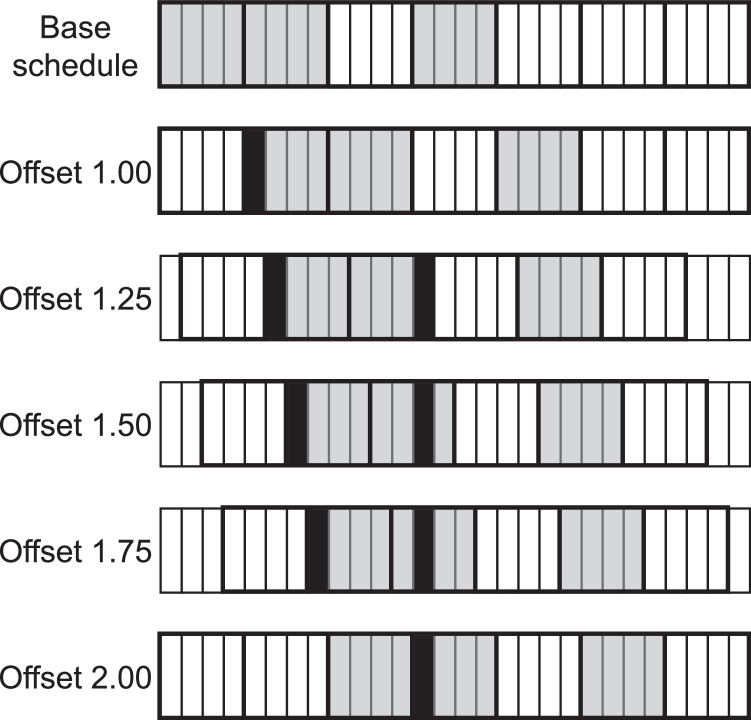
Illustration of the encounter opportunities yielded per cycle on a {7,3,1} block design considering 5 different offsets for *s* = 4 in a bidirectional discovery case. The black subslots indicate the encounter opportunities.

Even though the distinctive characteristic of the Projective Planes in comparison to other methods for generating asynchronous duty cycling schedules is the guarantee of yielding exactly 1 common active for non-zero integer offsets, Fig. 3 shows that they actually result in more encounter opportunities when one considers the proposed subslot-based methodology. The figure illustrates a full cycle of the base schedule (offset 0) followed by 5 selected rotations of the same schedule: for offsets 1, 1.25, 1.5, 1.75, and 2. In this figure, s=4 and we consider the bidirectional case. The black subslots denote the encounter opportunities.

For the integer non-zero offsets (1 and 2), as expected, there is exactly one encounter opportunity per cycle. Further notice that those opportunities happen at the second and third slots of the base schedule, respectively. However, each of the 3 non-integer offsets shown in the figure (1.25, 1.5, and 1.75) results in two encounter opportunities per cycle. That happens because a non-integer offset o can be seen as superposition of the integer offsets ⌊o⌋ and ⌈o⌉, *i.e.*, part of the overlapping active time occurs on the slot it would for the offset ⌊o⌋ while the other part occurs on the slot it would for the offset ⌈o⌉. That originates twice as many encounter opportunities for any non-integer offset o with 1<o<v. For non-integer offsets 0<o<1, there is a superposition between the offsets 0 and 1, which yields k encounters (due to the offset 0) plus another encounter (due to the offset 1).

Therefore, the expected number of encounters per cycle in the bidirectional discovery case for a {v,k,1} Projective Plane considering that each slot is divided into s subslots is given by:

$$E\left[e_{\left\{v,k,1\right\},s}\right]=k\cdot \frac{1}{sv}+1\cdot \frac{v-1}{sv}+\left(k+1\right)\cdot \frac{s-1}{sv}+2\cdot \frac{\left(s-1\right)\left(v-1\right)}{sv}$$

By increasing s, the model considers smaller subslots improving its temporal resolution and better capturing the continuous time of the real-world link. On the limit, when s→∞, we consider the continuous time case, and the expression simplifies to:

$$E\left[e_{\left\{v,k,1\right\}}\right]=\left(k+1\right)\cdot \frac{1}{v}+2$$

The NDT of a link between two nodes A and B following some asynchronous wakeup schedule can be written as a function of the probability p of a successful discovery when a node transmits a beacon and the receiver is active as follows:

$$NDT=\tau +\left(1-\frac{1}{p}\right)\cdot \frac{1}{E\left[e\right]}\cdot n,$$

where τ is the expected time until the first encounter opportunity, E[e] is the expected number of encounter opportunities per cycle and n is the length of the schedule in slots. For the case of a {v,k,1} Projective Plane, we can replace v for n and $E[e_{\left\{v,k,1\right\}}]$ for E[e], obtaining:

$$NDT=\tau +\left(1-\frac{1}{p}\right)\cdot \frac{1}{E\left[e_{\left\{v,k,1\right\}}\right]}\cdot v$$

Notice that for schedules with a large v – which correspond to lower duty cycle Projective Planes –, $E[e_{\left\{v,k,1\right\}}]$ is approximately 2, while the traditional slot-based methodology predicts 1 encounter per cycle. Further notice that, as p decreases, τ becomes less important and the NDT is dominated by the second term. That means that NDT under the subslot-based methodology should be roughly half of what is predicted by models based on the traditional slot-based methodology for lower values of p. Because there are closed-form formulas available in the literature to predict the NDT for Block Designs based on the slot-based methodology, those can be readily adapted to the subslot-based methodology for low values of p by simply dividing their values by 2.

Further notice that τ is also affected by the proposed methodology, since, with more encounter opportunities, the distance between two consecutive opportunities decreases and, thus, the expected time until the first encounter opportunity is reduced. However, mathematically modeling this reduction for the bidirectional discovery case is hard because it depends on the distribution of the two encounter opportunities for offsets ⌊o⌋ and ⌈o⌉ – for each non-integer offset o – which is outside the scope of this paper.

Conversely, in the unidirectional discovery case, the number of opportunities per cycle does not change. It is possible to see why by analyzing Fig. 3 again. Notice that, for the non-integer offsets shown in the figure, one of the encounter opportunities happens on the first subslot of a slot of the base schedule while the other happens on the first subslot of a slot of the rotated schedule. On the unidirectional discovery case, however, only one of those schedules would belong to the transmitter, meaning that only one of those would constitute an encounter opportunity. Thus, like in the traditional methodology, every offset o≥1 results in exactly one encounter opportunity while the other offsets result in k opportunities.

Interestingly, even though the expected number of encounter opportunities does not change in the unidirectional discovery case, the value of τ does. To see why, consider some pair of sensors A and B – A is the transmitter – operating under a certain Projective Plane with a relative offset o. Now, regardless of the particular value of o – and, therefore, of the number of encounter opportunities per cycle –, consider two consecutive encounter opportunities between A and B. Let d denote the distance, in slots, between those two encounter opportunities. Note that those opportunities must happen in the first subslot of certain slots of A and, therefore, d is integer. Further assume that A starts the discovery process in a moment between those two consecutive discovery opportunities (after the first, but perhaps exactly on the second). Under the traditional slot-based methodology, the expected number of slots until the first encounter opportunity is:

$$\bar{\tau_{trad}}\left[slots\right]=\frac{0+1+\cdots +\left(d-1\right)}{d}=\frac{d-1}{2}$$

However, under the proposed subslot-based methodology, time is counted in subslots and, therefore:

$$\bar{\tau_{prop}}\left[subslots\right]=\frac{0+1+\cdots +\left(ds-1\right)}{ds}=\frac{\left(ds-1\right)}{2}$$

Converting to slots:

$$\bar{\tau_{prop}}\left[slots\right]=\frac{d}{2}-\frac{1}{2s}$$

As s→∞, the second term vanishes and, therefore:

$$\bar{\tau_{prop}}\left[slots\right]=\frac{\left(d-1\right)}{2}$$

Since this can be generalized for any offset o and any pair of consecutive encounter opportunities, it follows that τ is half a slot larger under the subslot-based methodology when s→∞.

### Method validation

For validating the proposed subslot-based methodology, we used the numerical simulator described in the previous section and performed simulations considering different scenarios and parameter variations. Notice that when the number of subslots per slot s is 1, the subslot-based methodology is equivalent to the slot-based methodology. Each point shown in the following graphs corresponds to the average NDT considering 1000,000 repetitions of the corresponding simulation. All results include error bars denoting the respective 95 % confidence intervals.

**Fig. 4:**
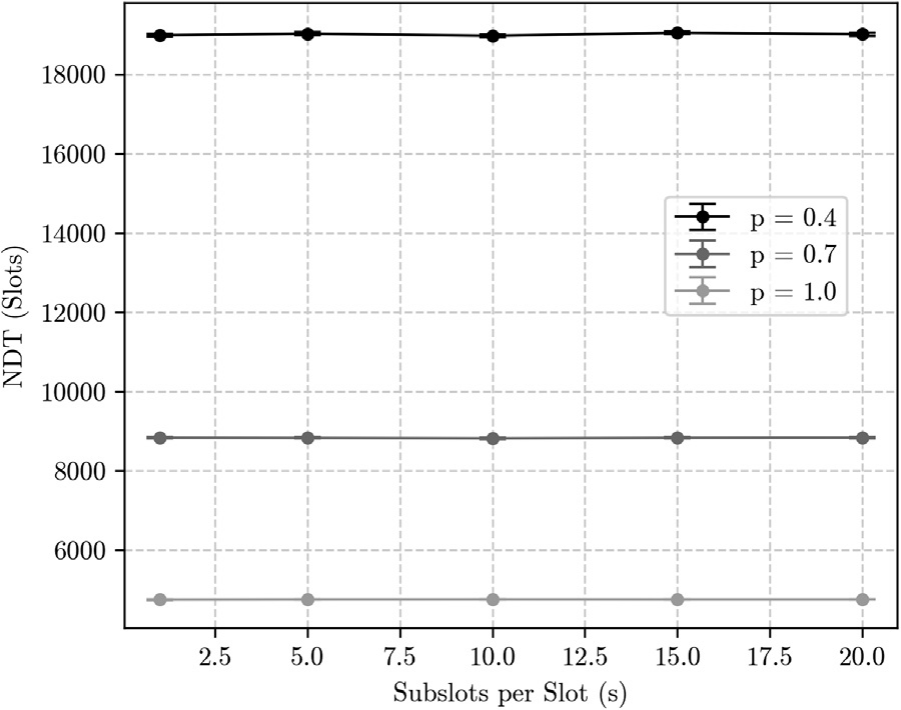
Simulation results for a {9507, 98, 1} block design under the unidirectional discovery scenario, considering different discovery success probabilities (p) and number of subslots per slot (s).

As a first experiment, we simulated a unidirectional discovery scenario using a {9507, 98, 1} Block Design varying the discovery success probability p and the number of subslots per slot s. Results are shown in Fig. 4. As predicted, NDT values do not change significantly between the slot-based (s=1) and the subslot-based methodologies (s>1), since the number of encounter opportunities is the same under both. Indeed, the only change predicted in this case is an increase of half a slot as s→∞, but that is not perceptible in these results due to the magnitude of the NDT and other sources of variability for such a low duty cycle schedule.

**Fig. 5:**
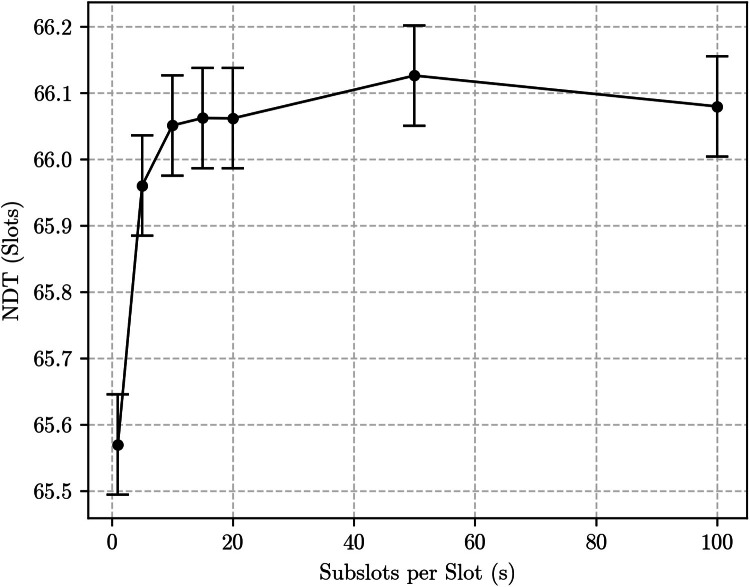
Simulation results for the unidirec6 and 6tional discovery case using the {133, 12, 1} Block Design with *p* = 1.

**Fig. 6:**
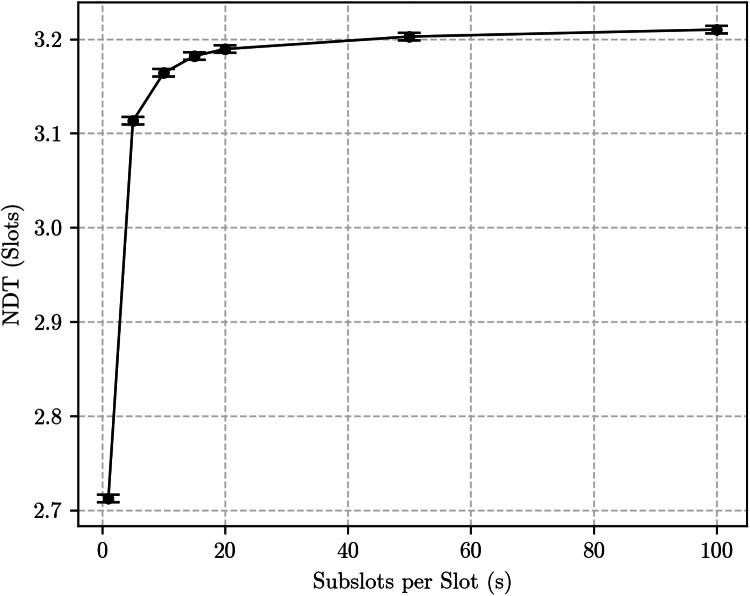
Simulation results for the unidirectional discovery case using the {7, 3, 1} block design with *p* = 1.

To better see and validate that half slot difference, we run simulations fixing p=1 (as to remove the variance introduced by the outcome of the beacon transmission) and using higher duty cycle Block Designs. Fig. 5 shows the results for the {133, 12, 1} Block Design – resulting in a 9.02 % duty cycle –, whereas Fig. 6 shows the results the {7, 3, 1} Block Design – with a 42.9 % duty cycle.

Both graphs (Fig. 5, Fig. 6) are qualitatively similar, albeit for very different values of NDT due to the significantly different duty cycle values. Because the {133, 12, 1} Block Design is also significantly longer, results have more statistical variability. Nevertheless, they both show a fast increase in NDT as s grows. For s=1 – the first point in each graph –, the NDT values match the predictions yielded by the slot-based methodology. For s=100, the resultant NDT has increased by almost half a slot, as expected.

**Fig. 7:**
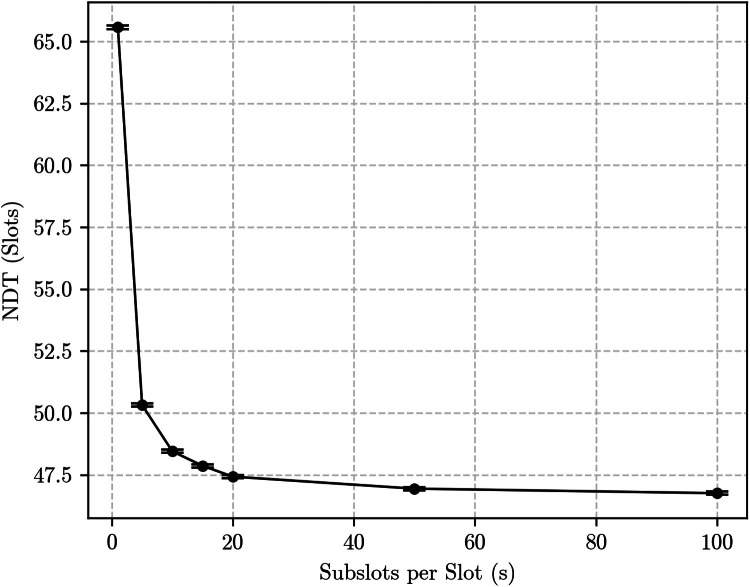
Simulation results for the bidirectional discovery case using the {133, 12, 1} block design with *p* = 1.

**Fig. 8:**
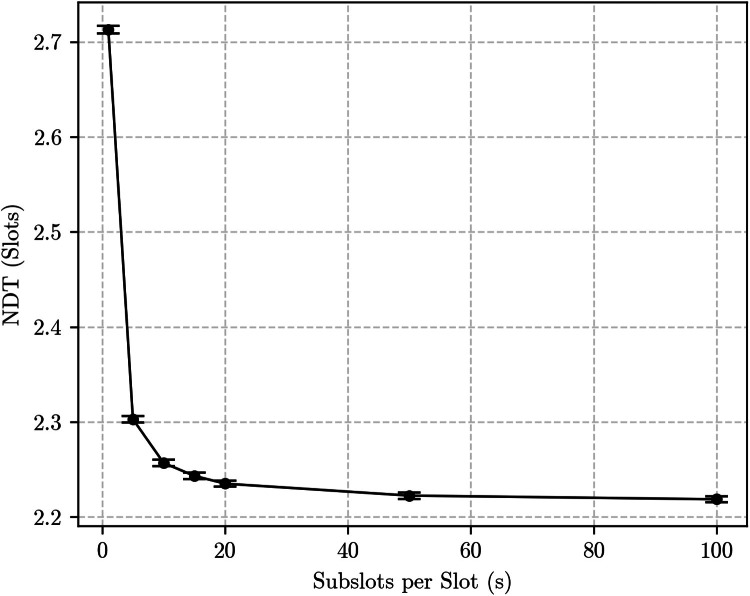
Simulation results for the bidirectional discovery case using the {7, 3, 1} block design with *p* = 1.

The bidirectional discovery case yields more substantial differences. For example, Fig. 7, Fig. 8 show simulation results for the same {133, 12, 1} and {7, 3, 1} Block Designs with p=1, but now in the bidirectional discovery scenario. The curves are, again, qualitatively similar: both show an exponential decrease of the NDT as s grows. This is expected, as the number of encounter opportunities roughly doubles as s→∞, decreasing the distance between consecutive encounter opportunities. However, the impact of that on the NDT is substantially different for each Block Design: for the {7, 3, 1}, it caused a reduction of approximately half a slot, or about 18 %, while for the {133, 12, 1} the reduction was of about 20 slots, which corresponds to roughly 30.3 %.

As one final validation, we ran simulations in the bidirectional discovery case with the {133, 12, 1} Block Design, but now considering a very low probability p=0.1. The purpose here is to validate that the expected number of encounter opportunities per cycle roughly doubles, as expected.

**Fig. 9:**
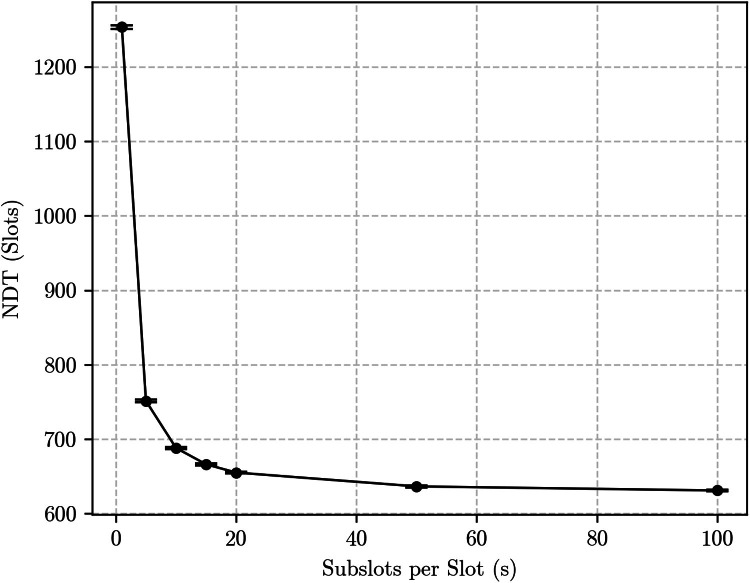
Simulation results for the bidirectional discovery case using the {133, 12, 1} block design with *p* = 0.1.

Fig. 9 shows the results. Once again, the NDT decreases exponentially as s→∞, but here the curve asymptotically approaches half the value measured at s=1, as predicted by the subslot-based methodology.

### Limitations

While the subslot-based methodology is able to more closely represent the behavior of a link operating under an asynchronous duty cycling schedule, it still has limitations. One of those is capturing the behavior of collisions. For example, in the bidirectional discovery case, when the offset o=0, the first subslots of the active slots in both nodes are perfectly aligned, which means both nodes would try to transmit at the exact same time, causing a collision. In this work, we did not take that into account, which can skew the results. It is also important to study how transmission and propagation delays affect the proposed methodology, particularly when modeling a link for which the discovery protocol involves multiple frames (*e.g.*, a beacon followed by an ack).

## Ethics statements

This work did not involve human subjects, animal experiments data, or data collected from social media platforms.

## CRediT authorship contribution statement

**Diego Passos:** Conceptualization, Methodology, Software, Formal analysis, Writing – original draft. **Beatriz Trabbold:** Investigation, Writing – original draft. **Ricardo C. Carrano:** Conceptualization, Methodology, Writing – review & editing, Supervision. **Cledson de Sousa:** Conceptualization, Validation, Writing – review & editing.

## Declaration of competing interest

The authors declare that they have no known competing financial interests or personal relationships that could have appeared to influence the work reported in this paper.

## Data Availability

Data will be made available on request.
